# Novel serum metabolites associate with cognition phenotypes among Bogalusa Heart Study participants

**DOI:** 10.18632/aging.102107

**Published:** 2019-07-21

**Authors:** Mengyao Shi, Lydia A. Bazzano, Jiang He, Xiaoying Gu, Changwei Li, Shengxu Li, Kristine Yaffe, Jason M. Kinchen, Patrick Stuchlik, Xuenan Mi, Jovia L. Nierenberg, Alexander C. Razavi, Tanika N. Kelly

**Affiliations:** 1 Department of Epidemiology, Tulane University, New Orleans, LA 70112, USA; 2 Institute of Clinical Medical Science, China-Japan Friendship Hospital, National Clinical Research Center of Respiratory Diseases, Beijing, China; 3 Department of Epidemiology and Biostatistics, University of Georgia, Athens, GA 30602, USA; 4 Children's Minnesota Research Institute, Children's Hospitals and Clinics of Minnesota, Minneapolis, MN 55404, USA; 5 School of Medicine, University of California San Francisco, San Francisco, CA 94143, USA; 6 Metabolon, Inc., Durham, NC 27560, USA

**Keywords:** metabolomics, metabolite network, dementia, Alzheimer’s disease, cognition

## Abstract

Background: Metabolomics study provides an opportunity to identify novel molecular determinants of altered cognitive function.

Methods: During 2013 to 2016 Bogalusa Heart Study (BHS) visit, 1,177 participants underwent untargeted, ultrahigh performance liquid chromatography-tandem mass spectroscopy metabolomics profiling. Global cognition and five cognition domains were also assessed. The cross-sectional associations of single metabolites with cognition were tested using multiple linear regression models. Weighted correlation network analysis was used to examine the covariable-adjusted correlations of modules of co-abundant metabolites with cognition. Analyses were conducted in the overall sample and according to both ethnicity and sex.

Results: Five known metabolites and two metabolite modules robustly associated with cognition across overall and stratified analyses. Two metabolites were from lipid sub-pathways including fatty acid metabolism [9-hydroxystearate; minimum P-value (min-P)=1.11×10^-5^], and primary bile acid metabolism (glyco-alpha-muricholate; min-P=4.10×10^-5^). One metabolite from the glycogen metabolism sub-pathway (maltose; min-P=9.77×10^-6^), one from the polyamine metabolism sub-pathway (N-acetyl-isoputreanine; min-P=1.03×10^-5^), and one from the purine metabolism sub-pathway (7-methylguanine; min-P=1.19×10^-5^) were also identified. Two metabolite modules reflecting bile acid metabolism and androgenic steroids correlated with cognition (min-P=5.00×10^-4^ and 3.00×10^-3^, respectively).

Conclusion: The novel associations of 5 known metabolites and 2 metabolite modules with cognition provide insights into the physiological mechanisms regulating cognitive function.

## Introduction

Dementia affects 43.8 million adults worldwide [1]. Due to increasing longevity globally, the absolute number of individuals living with dementia is expected to triple by 2050 [2]. Alzheimer’s disease (AD) is the most common type of dementia, representing 70% of all dementia cases and affecting approximately 5 million U.S. adults [3]. As the country’s sixth leading cause of death and a leading cause of disability and poor health, AD represents a major public health challenge [4]. Despite the well-established burden of AD on individual patients, their caregivers, and society, there are few effective strategies for the early prevention and treatment of this debilitating condition.

The long prodromal period preceding AD, which may include over a decade of pre-clinical cognitive decline followed by mild cognitive impairment (MCI) [5,6], provides an opportunity to identify novel molecular precursors to clinical symptoms and diagnosis of AD. Indeed, previous studies have discovered various blood metabolites that can differentiate normal controls from those MCI, in addition to AD [7–11]. Although these findings are promising, the previous studies have been limited in sample size or to the sole use of targeted metabolomics approaches which only measure pre-specified, biological candidate metabolites [7–11]. To our knowledge, only one previous study employed agnostic untargeted metabolomics profiling to discover novel metabolites for early cognitive decline [12]. Additional research in this area is critically needed to identify novel biomarkers for early AD prediction and potential targets for molecular-based interventions aimed at AD prevention and early treatment.

In the current study, we aimed to identify novel serum metabolites and metabolite networks associated with cognition in middle-aged adults, prior to any clinical symptoms. Our analysis leveraged data collected from the large, biracial Bogalusa Heart Study (BHS), whose participants underwent untargeted, ultrahigh performance liquid chromatography-tandem mass spectroscopy metabolomics profiling and were carefully phenotyped for multiple domains of cognition, along with important covariables, at the recently completed 2013 to 2016 study visit.

## RESULTS

Characteristics of the 1,177 BHS metabolomics study participants are shown in Table 1. On average, BHS participants were middle-aged, obese, with systolic BP, glucose, and LDL-C values elevated slightly above the normal range. The participants were predominantly female, non-smokers, and approximately half were current drinkers and had at least a high-school education. As expected, BHS participants tended to perform well on all eight tests of cognitive function, which measured global cognition as well as the domains of verbal memory, attention and concentration, processing speed, ability to decode, and executive function (Table 1 and Supplementary Figure 1).

**Table 1:** Characteristics of BHS Participants (n=1,177).

**Characteristic**	**Overall**	**White Male (n=324)**	**White Female (n=449)**	**Black Male (n=150)**	**Black Female (n=254)**
Age(years), mean (SD)	48.11 (5.26)	48.92 (4.91)	48.15 (5.08)	47.27 (6.03)	47.53 (5.38)
<=12 years (high school), n (%)	589 (50.04%)	156 (48.15%)	176 (39.20%)	105 (70.00%)	152 (59.84%)
Vocabulary score, mean (SD)	26.54 (9.83)	28.87 (8.94)	30.43 (9.30)	20.23 (7.88)	20.42 (7.91)
Depression, n (%)	124 (10.54%)	26 (8.02%)	60 (13.36%)	11 (7.33%)	27 (10.63%)
Smoking, n (%)					
Never	600 (50.98%)	158 (48.77%)	243 (54.12%)	50 (33.33%)	149 (58.66%)
Former	348 (29.57%)	105 (32.41%)	133 (29.62%)	47 (31.33%)	63 (24.80%)
Current	229 (19.46%)	61 (18.83%)	73 (16.26%)	53 (35.33%)	42 (16.54%)
Drinking, n (%)					
Never	138 (11.72%)	13 (4.01%)	55 (12.25%)	17 (11.33%)	53 (20.87%)
Former	379 (32.20%)	109 (33.64%)	145 (32.29%)	48 (32.00%)	77 (30.31%)
Current	660 (56.07%)	202 (62.35%)	249 (55.46%)	85 (56.67%)	124 (48.82%)
BMI (kg/m^2^), mean (SD)	31.37 (7.79)	30.46 (6.05)	30.12 (7.40)	31.13 (8.58)	34.87 (8.88)
SBP (mmHg), mean (SD)	123.08 (16.72)	125.35 (13.69)	117.28 (14.45)	131.50 (15.86)	125.46 (20.68)
Glucose(mg/dl), mean (SD)	106.07 (34.64)	106.90 (26.73)	103.88 (35.65)	107.55 (31.81)	108.03 (42.47)
LDL cholesterol(mg/dl), mean (SD)	114.61 (35.06)	116.97 (33.74)	116.04 (33.91)	108.02 (35.82)	112.95 (37.81)
Global cognition, median (IQR)	0.52 (7.68)	0.71 (6.91)	2.98 (6.80)	-3.80 (6.39)	-1.36 (7.32)
Verbal Memory					
Logical memory I, median (IQR)	20.00 (10.00)	20.00 (9.00)	22.00 (9.00)	17.00 (10.00)	18.00 (8.00)
Logical memory II, median (IQR)	16.00 (10.00)	16.00 (10.00)	18.00 (9.00)	12.00 (8.00)	14.00 (8.00)
Logical memory II-recognition, median (IQR)	24.00 (4.00)	24.00 (4.00)	25.00 (4.00)	22.00 (5.00)	23.00 (4.00)
Attention and Concentration					
Digit span forward, median (IQR)	11.00 (4.00)	12.00 (4.00)	12.00 (4.00)	10.50 (4.00)	11.00 (4.00)
Digit span backward, median (IQR)	7.00(3.00)	8.00 (4.00)	8.00 (4.00)	6.00 (3.00)	7.00 (2.00)
Processing Speed					
Digit coding, median (IQR)	60.00 (24.00)	57.00 (20.00)	67.00 (23.00)	45.00 (20.00)	59.00 (25.00)
Trial making test A, median (IQR)	0.42 (0.21)	0.43 (0.20)	0.39 (0.18)	0.48 (0.25)	0.44 (0.21)
Ability to Decode					
Word reading, median (IQR)	42.00 (13.00)	45.00 (10.00)	46.00 (9.00)	36.00 (16.00)	37.00 (13.00)
Executive function					
Trial making test B, median (IQR)	0.91 (0.53)	0.89 (0.48)	0.83 (0.44)	1.16 (0.67)	1.01 (0.57)

Data are presented as mean (standard deviation) or median (interquartile range) for continuous variables and as percentage for categorical variables.

BMI: body mass index

SBP: systolic blood pressure

LDL: low-density lipoprotein

SD: standard deviation

IQR: interquartile range

### Association of single metabolites with cognitive tests

**Figure 1:**
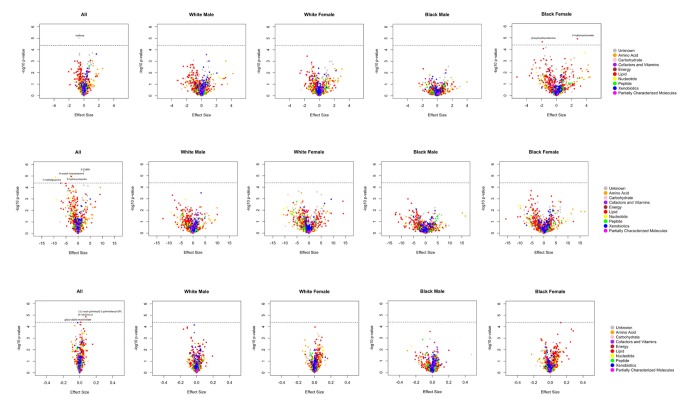
**A. Volcano plots of effect sizes versus –log10 P values for all 1202 metabolites among BHS participants, according to cognitive domain.** (**A**) Global cognition; (**B**) Processing speed (digit coding test); (**C**) Processing speed (trail making test A).

A total of 14 metabolites achieved Bonferroni corrected significance in the overall and/or ethnicity-sex stratified analyses (Figure 1 and Supplementary Figure 2). Among them, 6 metabolites were robustly association with cognition phenotypes, demonstrating consistent effect directions across all analyses. These metabolites included 5 known biochemicals (Table 2) and 1 still unrecognized biochemicals (Supplementary Table 1). One of these metabolites, maltose, was negatively associated with global cognition score [minimum P-value (min-P) =9.77E-06]. Five metabolites were associated with the processing speed cognitive domain. Among them, four metabolites were negatively associated with digit coding test scores, including: N-acetyl-isoputreanine which belongs to the amino acid super pathway (min-P =1.03E-05), 9-hydroxystearate which belongs to the lipid super pathway (min-P =1.11E-05), 7-methylguanine which belongs to the nucleotide super pathway (min-P =1.19E-05), and unknown metabolite X-21840 (min-P =4.32E-06). From the lipid super pathway, the remaining metabolite, glyco-alpha-muricholate (min-P =4.10E-05), was positively associated with trail making test A scores. The remaining 8 metabolites, which reached Bonferroni-corrected significance in the overall or either ethnicity-sex-specific analysis, had inconsistent effect directions across the groups (Supplementary Table 2). Although not significant by the stringent criteria used in the current study, these promising metabolites included two associated with the global cognition domain (3-hydroxyoctanoate and phosphoethanolamine), one associated with the verbal memory domain (3-methoxytyrosine), two associated with the attention & concentration domain [1-palmitoyl-GPC (16:0) and 1-palmitoyl-2-stearoyl-GPC (16:0/18:0)], one associated with the processing speed domain [1-(1-enyl-palmitoyl)-2-palmitoleoyl-GPC (P-16:0/16:1)], and two associated with the executive function domain [methionine sulfone and 1-stearoyl-2-oleoyl-GPC (18:0/18:1)].

**Table 2:** Novel metabolites achieving significance in BHS participants.

**Super Pathway**	**Sub Pathway**	**Metabolite**	**BHS Sample**	**ES**	**SE**	**P**
Global Cognition						
Carbohydrate	Glycogen Metabolism	Maltose^1^	Overall	-0.67	0.15	9.77E-06
			White Male	-0.65	0.25	9.66E-03
			White Female	-1.01	0.31	1.12E-03
			Black Male	-0.47	0.35	1.76E-01
			Black Female	-0.07	0.38	8.60E-01
Processing Speed						
Amino Acid	Polyamine Metabolism	N-acetyl-isoputreanine*^2^	Overall	-3.11	0.70	1.03E-05
			White Male	-0.95	1.23	4.41E-01
			White Female	-3.66	1.32	5.71E-03
			Black Male	-2.47	1.55	1.15E-01
			Black Female	-4.82	1.85	9.77E-03
Lipid	Fatty Acid, Monohydroxy	9-hydroxystearate^2^	Overall	-2.79	0.63	1.11E-05
			White Male	-1.44	1.17	2.18E-01
			White Female	-2.35	0.99	1.82E-02
			Black Male	-3.61	1.76	4.23E-02
			Black Female	-5.06	1.69	2.98E-03
Nucleotide	Purine Metabolism, Guanine containing	7-methylguanine^2^	Overall	-9.46	2.15	1.19E-05
			White Male	-9.14	3.78	1.61E-02
			White Female	-10.55	3.91	7.18E-03
			Black Male	-8.42	5.29	1.14E-01
			Black Female	-5.89	5.12	2.51E-01
Lipid	Primary Bile Acid Metabolism	Glyco-alpha-muricholate**^3^	Overall	0.01	0.0032	4.10E-05
			White Male	0.01	0.0045	1.58E-02
			White Female	0.02	0.01	1.27E-03
			Black Male	0.01	0.01	1.54E-01
			Black Female	0.01	0.01	2.19E-01

ES=Effect size; SE=Standard error. Adjusted for age, gender, ethnicity, cigarette smoking, drinking, education, depression, vocabulary, BMI, SBP, LDL-C and glucose.

* Indicates compounds that have not been officially confirmed based on a standard, but we are confident in its identity.

** Indicates a compound for which a standard is not available, but we are reasonably confident in its identity.

1. Associated with global cognition score.

2. Associated with digit coding test.

3. Associated with trail making test A.

**Figure 2:**
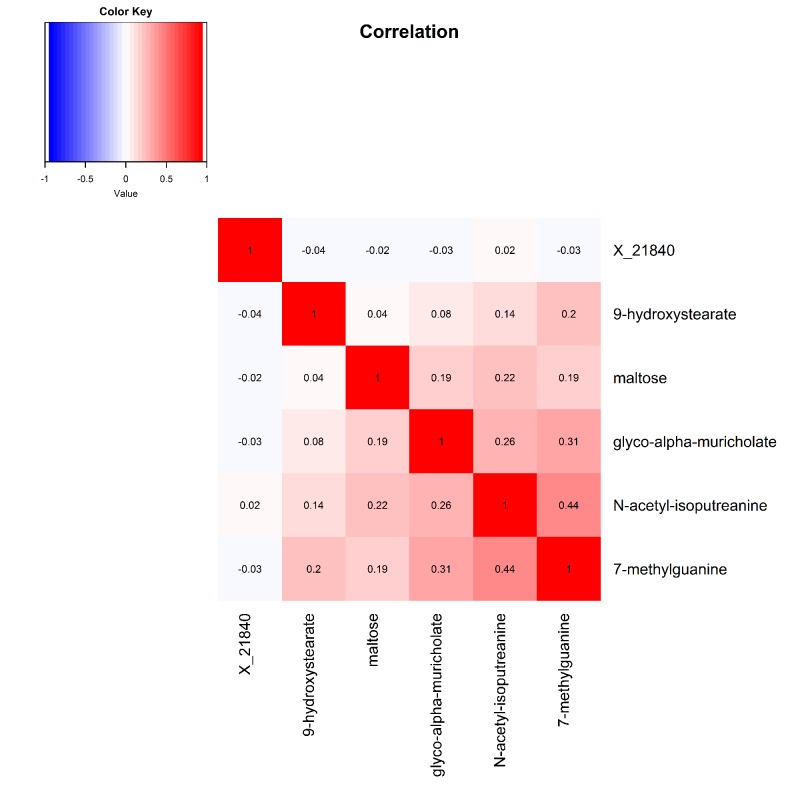
Heat map displaying pairwise correlation coefficients for the six identified metabolites.

Pearson correlations between the six robustly identified metabolites are presented in Figure 2. Metabolites associated with digit coding and trail making test A, which both measure the processing speed cognitive domain, were all modestly to moderately correlated, with correlation coefficients ranging from 0.08 to 0.44.

### Associations of metabolite modules with cognitive tests

The 9 metabolite modules identified among BHS participants are depicted in Figure 3A, Figure 3B, Figure 3C and Supplementary Figure 3. A module comprised of metabolites involved in primary and secondary bile acid metabolism were consistently associated with processing speed. This module demonstrated negative associations with digit coding test scores (min-P =6.00E-04), where higher test score values represent better test performance, and positive associations with TMT A scores (min-P =5.00E-04), where lower test score values represent better test performance. The network of eight metabolites most highly correlated with this module’s eigenmetabolite value (r>0.70) are presented in Figure 4. Additionally, a module comprised of metabolites involved in androgenic steroids was associated with global cognition score. This module demonstrated positive associations with global cognition score (min-P =3.00E-03), where higher test score values represent better test performance. The network of seven metabolites most highly correlated with this module’s eigenmetabolite value (r>0.70) are presented in Figure 5.

**Figure 3A:**
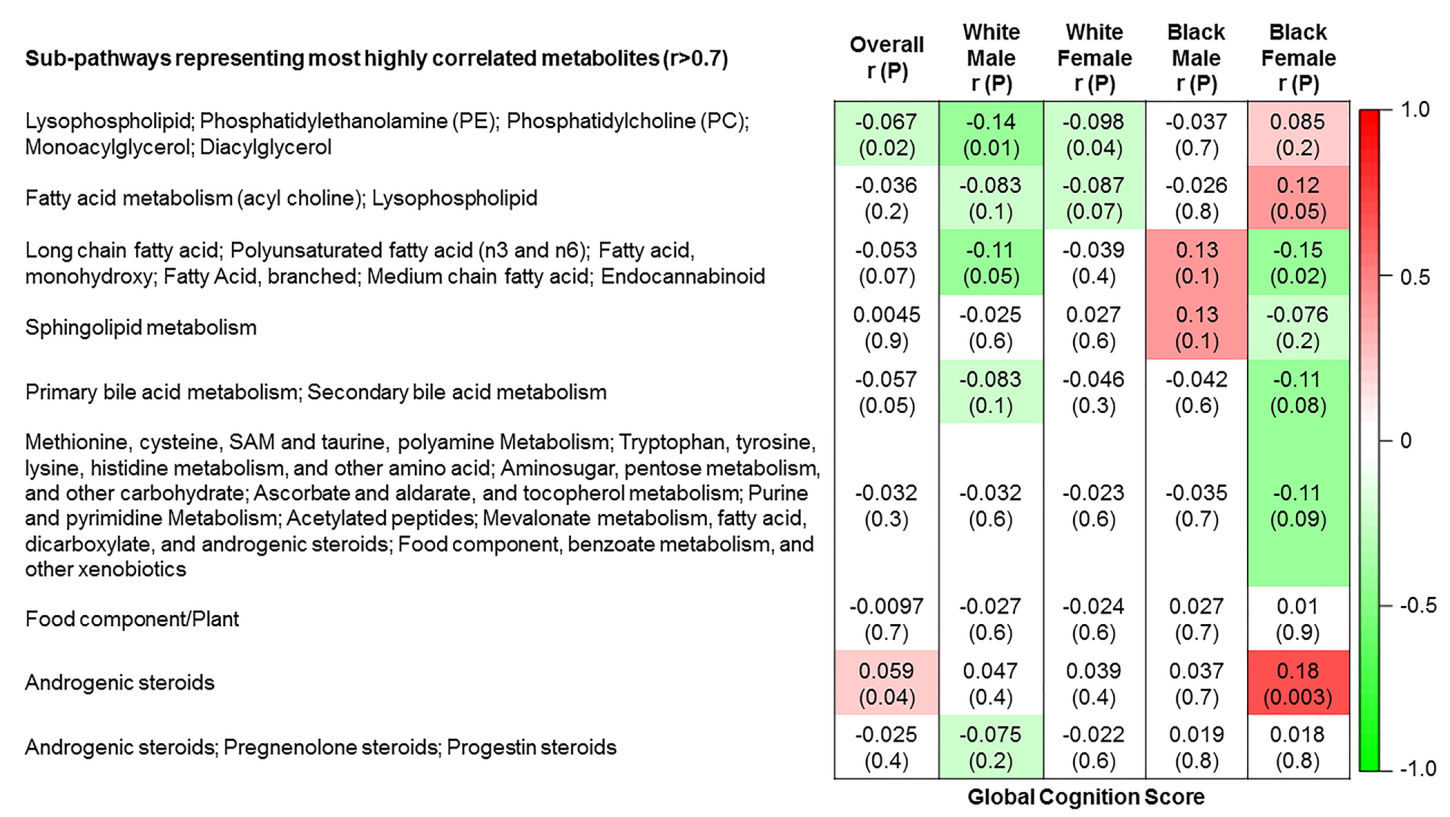
**Correlations of metabolite modules with cognition.** Global cognition (global cognition score).

**Figure 3B:**
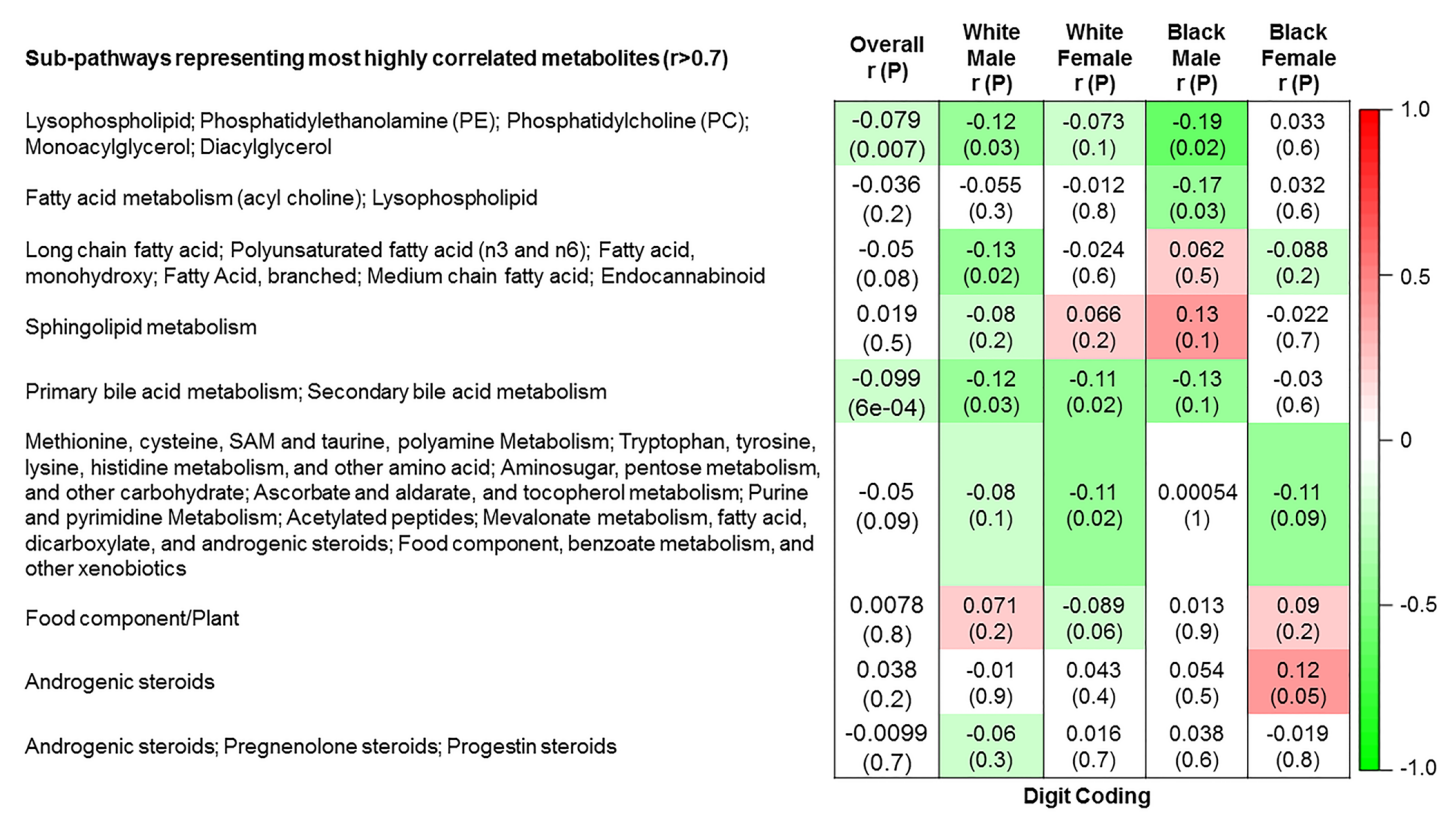
**Correlations of metabolite modules with cognition.** Processing speed domain (digit coding test).

**Figure 3C:**
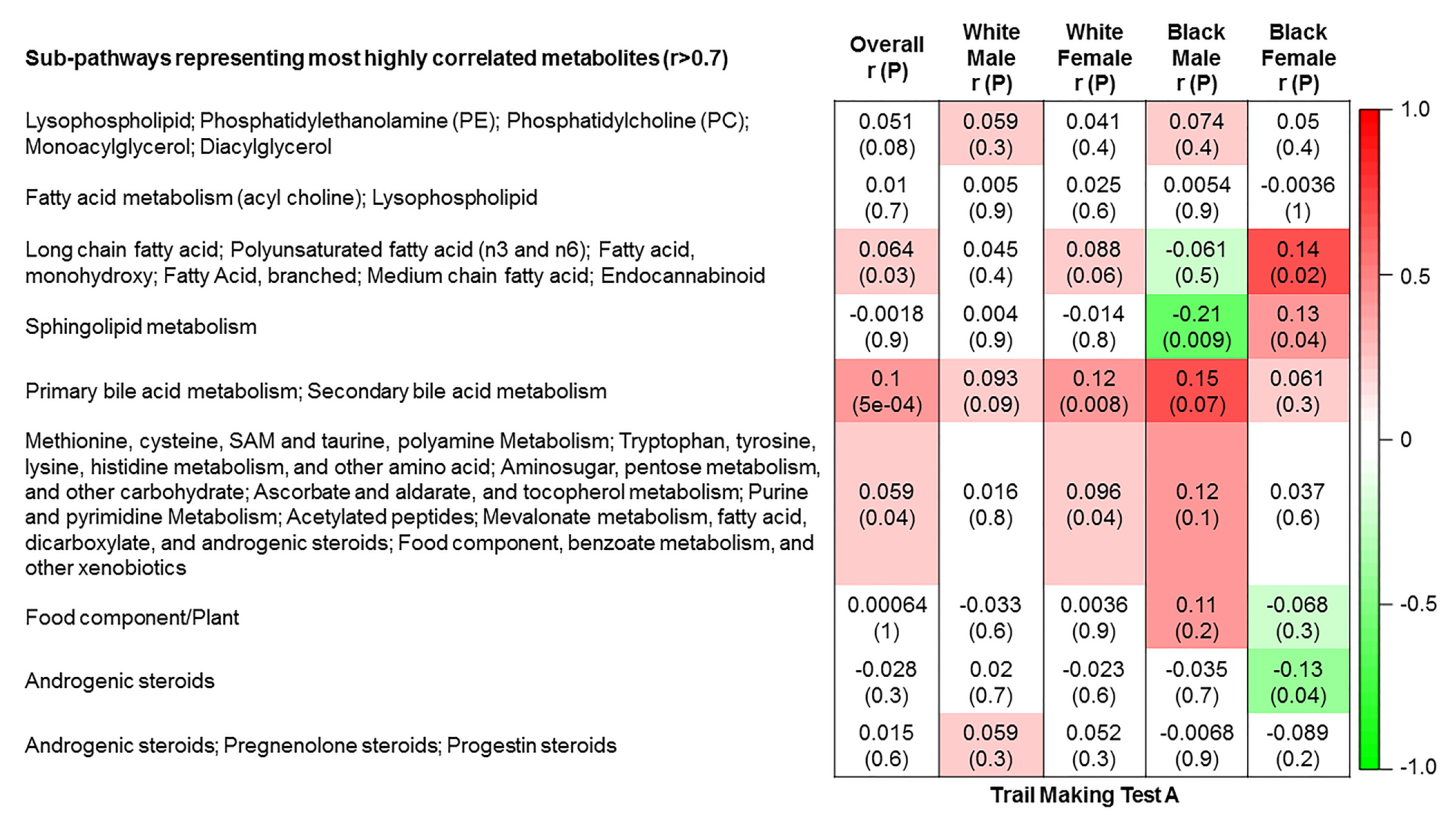
**Correlations of metabolite modules with cognition.** Processing speed domain (trail making test A).

**Figure 4:**
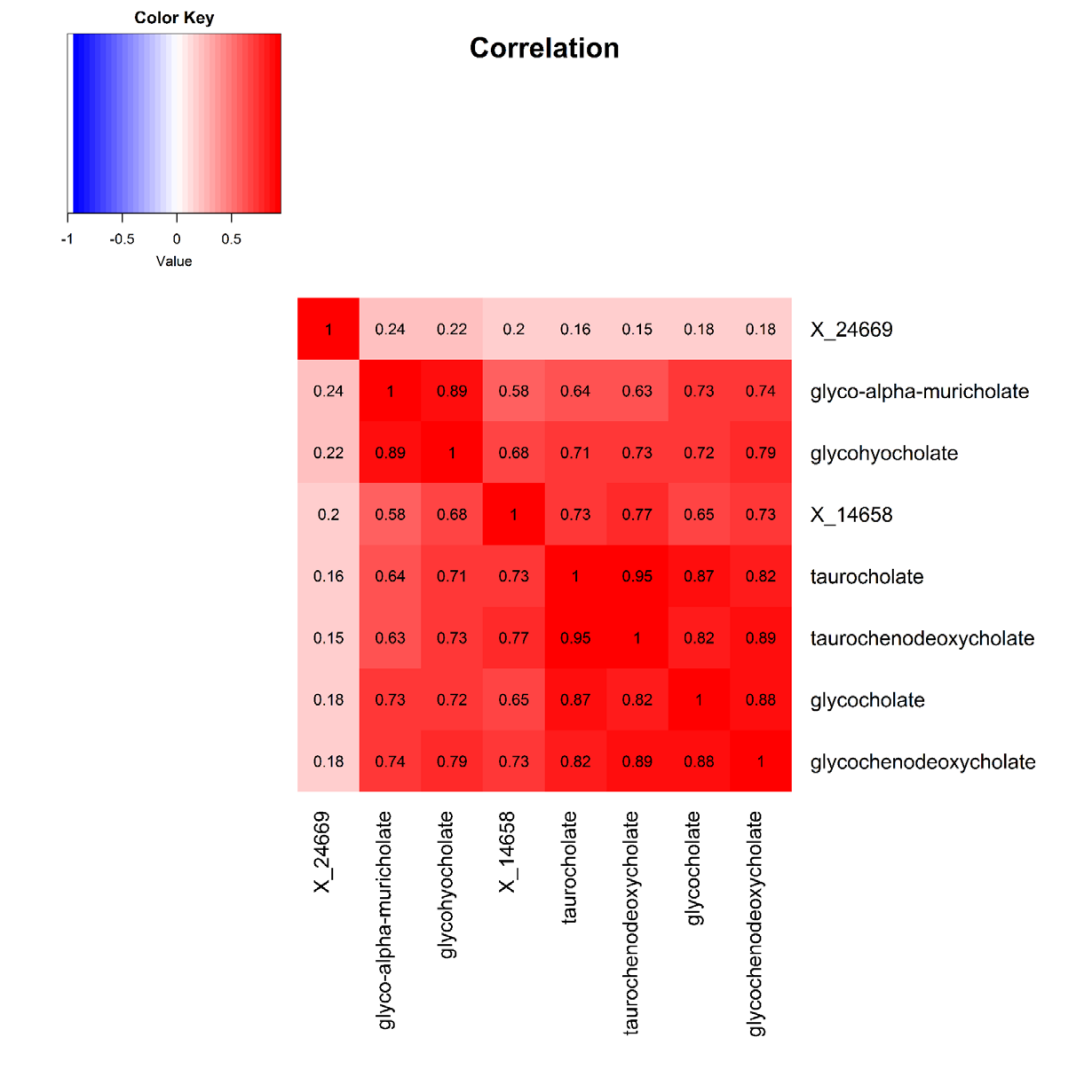
Heat map displaying pairwise correlation coefficients for the network of metabolites representing the significant primary and secondary bile acid metabolism pathway.

**Figure 5:**
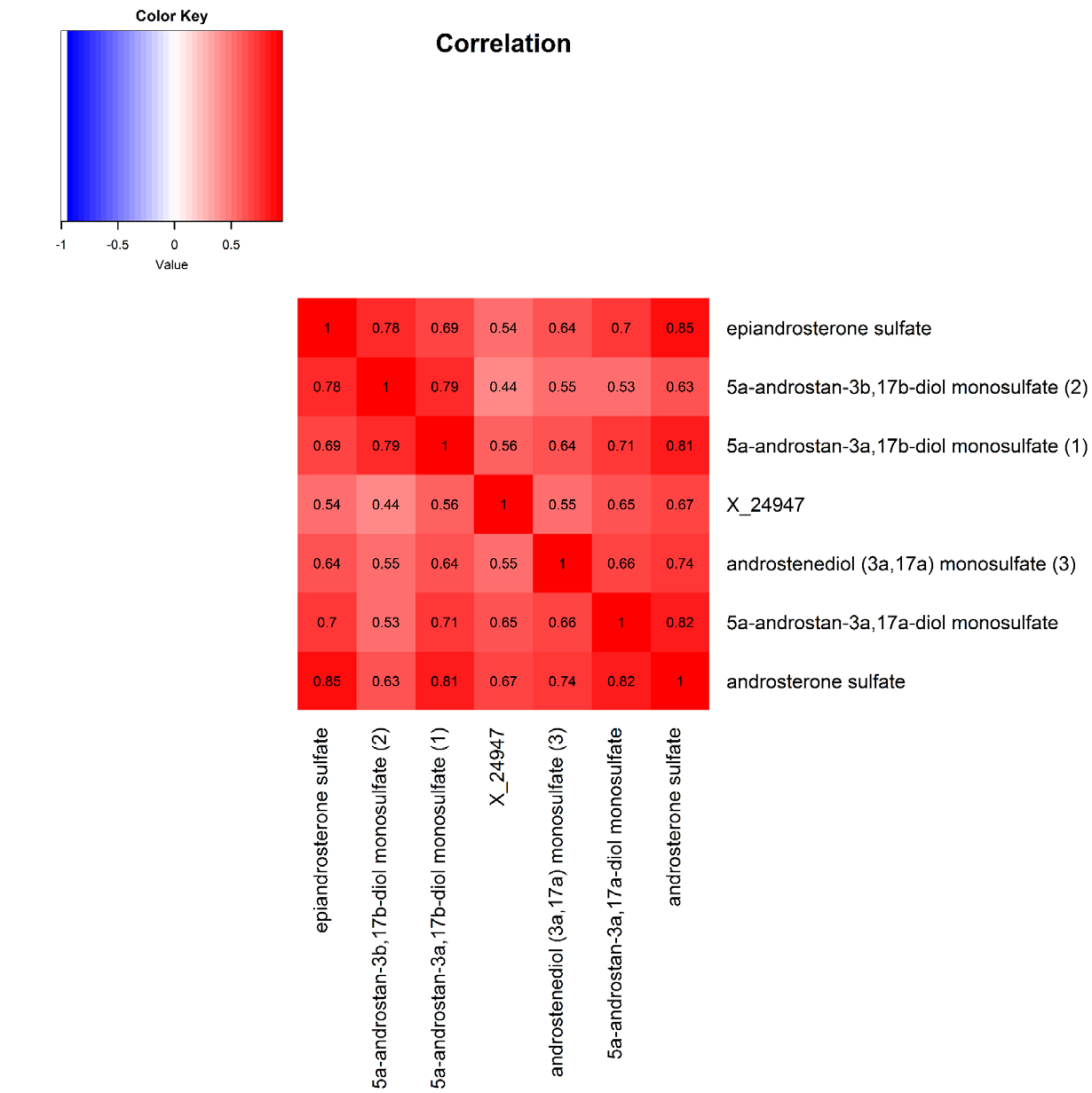
Heat map displaying pairwise correlation coefficients for the network of metabolites representing the significant androgenic steroids pathway.

## DISCUSSION

The current study robustly identified novel associations of six individual metabolites and two metabolite modules with cognition phenotypes among BHS participants. Identified metabolites included 5 known biochemical compounds along with 1 still unidentified analyte. Among the 5 known metabolites, one involved in glycogen metabolism, maltose, was associated with global cognition. An additional four metabolites associated with the processing speed cognitive domain including: N-acetyl-isoputreanine, an amino acid involved in polyamine metabolism; 9-hydroxystearate, a mono-hydroxy fatty acid metabolite; 7-methylguanine, a nucleotide involved in guanine containing purine metabolism; and glyco-alpha-muricholate, a metabolite involved in primary bile acid metabolism. Bolstering findings from the single metabolite analysis, a correlated network of metabolites involved in primary and secondary bile acid metabolism, which included glycol-alpha-muricholate, consistently associated with processing speed. In addition, one metabolite module comprised of metabolites related to androgenic steroids, which were not identified in the single metabolite analyses, were correlated with global cognition score. In aggregate, these findings identify several novel biomarkers of cognitive function in middle-aged adults, prior to clinical symptoms and onset of AD.

One single metabolite, maltose, and one metabolite module comprised of androgenic steroid related metabolites associated with global cognition in the current analysis. As one of the major disaccharides, maltose is produced by the breakdown of starch and can be further metabolized into two glucose molecules [13]. Similar to glucose, serum insulin concentrations have been empirically demonstrated to increase in response to intravenous maltose infusion [14–16], with maltose providing twice the calories for the same volume and concentration of glucose [14,15]. While insulin resistance and obesity, in the context of the metabolic syndrome, have been independently linked to cognitive decline [17,18], our study demonstrated a persistent inverse relation between maltose and cognitive phenotypes even after adjustment for BMI, systolic BP, and fasting LDL-C and glucose levels. While future research in this area is needed, these data suggest potentially novel biological pathways linking maltose to cognition. In addition to maltose, network analyses suggested a role for androgenic steroid metabolites in global cognition. Sex differences in cognitive function and certain neuropsychiatric diseases have been reported previously [19–21]. Consistent with findings reported here, sex hormones, including androgens, were believed to be important drivers underlying these associations [20,22]. Our findings add to the previous literature, suggesting that within each sex group and after adjusting for sex, androgenic steroid metabolite levels directly associate with cognition.

Four correlated metabolites and one metabolite module associated with the processing speed cognitive domain in our analyses. Among them, three metabolites associated with the digit coding test and included N-acetyl-isoputreanine, 9-hydroxystearate and 7-methylguanine. N-acetyl-isoputreanine is a purported byproduct of aldehyde dehydrogenase (ALDH) enzymatic action and end-product of polyamine metabolism [23]. ALDH is an enzyme critical to alcohol metabolism while polyamine metabolism plays an important role in cell growth and differentiation. Since both ALDH activity and polyamine metabolism have been implicated in cognitive dysfunction, the results of this analysis suggest a potentially shared biological mechanism of action [24–30]. As an endogenous lipoperoxidation product, 9-hydroxystearate is poorly studied but its parent compound, 9-hydroxystearic acid, is a well-known negative regulator of histone deacetylase (HDAC) [31,32]. HDAC can remove the acetyl group from histone proteins, thereby decreasing gene transcription rates [33]. Histone acetylation levels have previously been linked to cognitive dysfunction [33–35], and HDAC inhibitors are considered promising future therapeutic agents for the treatment of AD [34]. While we are the first to link 7-methylguanine to a complex cognition phenotype, this metabolite has previously been associated with Huntington’s disease (HD), a monogenic disorder with profound effects on cognitive and motor function [36]. Furthermore, urine levels of 7-methylguanine are a well-known indicator of tobacco smoking, which has been previously linked to cognitive decline and may suggest a novel biological mechanism mediating this relation [37–39]. In aggregate, the potential biological relevance of these metabolites to cognition strongly support a need further study of their temporal relations to cognitive decline.

The remaining metabolite associated with processing speed was identified by the TMT A test. The recently identified glyco-alpha-muricholate metabolite belongs to the primary bile acid metabolism sub-pathway, which is essential for the digestion of dietary fats and the secretion of lipids [40]. Bolstering the evidence for its role in cognition was our observation of a network of correlated primary and secondary bile acid metabolism metabolites, including glycol-alpha-muricholate, that collectively and consistently associated with processing speed as measured by both the digit coding and TMT A assessments. Furthermore, animal models have demonstrated the therapeutic benefits of tauroursodeoxycholic acid, an endogenous bile acid, in both AD and HD pathologies [41,42]. In humans, altered bile acid profiles have also been reported in AD [43], with our findings further adding evidence of its potentially important role in the earlier regulation of cognition.

The current study has several strengths. To our knowledge, this is the largest untargeted metabolomics study of cognition conducted to date. The large sample size enabled analyses stratified by both ethnicity and sex, allowing us to report 6 metabolites that may be relevant to diverse populations. Furthermore, metabolomics profiling, covariable measurement, and cognition phenotyping was conducted using a stringent study protocol with rigorous quality assurance and quality control procedures employed. Certain limitations should also be mentioned. As a cross-sectional study, this analysis cannot establish a temporal relationship between the identified metabolites and cognition phenotypes. Thus, prospective studies of these metabolites are needed to assess the etiologic relevance of our findings. To minimize false positive findings, only metabolites with consistency in effect direction across the mutually exclusive ethnic and sex groups were considered robustly significant in the current analysis. However, this rigorous control of type 1 error may have limited our ability to detect metabolites with effects that are relevant to specific populations. The eight metabolites that achieved Bonferroni corrected significance but lacked consistency in effect directions across ethnic- or sex-groups warrant confirmation by future studies.

The current study identified novel associations of five metabolites of known biochemical structure and two metabolite modules with cognition. Although we are the first to describe these metabolite-cognition signals, the biological pathways represented by the identified metabolites generally demonstrated clear relevance to cognition, providing additional qualitative support of our findings. In aggregate, this study offers new insights into the molecular mechanisms regulating cognitive function. Furthermore, the metabolites reported here should be evaluated for their longitudinal relationships with cognitive decline and development of MCI and dementia.

## MATERIALS AND METHODS

### Study population

The BHS is a community-based long-term study investigating the natural history of cardiovascular disease among a biracial sample (65% white and 35% African-American) of residents from Bogalusa, Louisiana, begun in 1973 by Dr. Gerald Berenson. From 1973 to today, 7 surveys were conducted in children and adolescents aged 4 to 17 years, and 11 surveys were conducted among adults aged 18 to 51 years who had been examined previously as children. The current BHS cohort includes 1,298 participants born between 1959 and 1979 who were screened at least 2 times during childhood and 2 times during adulthood for cardiovascular disease risk factors. Data and specimens collected in the 2013 to 2016 follow-up visit were used in cross-sectional analysis of these participants. Among the 1,298 eligible participants, those missing metabolomics (n = 37), covariable (n=80), or cognition test data (n=20) were excluded, leaving 1,177 participants for the study.

Informed consents were obtained from all the Bogalusa Heart Study participants after detailed explanation of the study. The study was approved by the Institutional Review Board at Tulane University.

### Metabolite profiling

Untargeted, ultrahigh performance liquid chromatography-tandem mass spectroscopy (UPLC-MS/MS) was conducted by Metabolon© using BHS serum samples that had been stored at -80°C since the 2013 to 2016 visit [44]. Rigorous quality assurance was conducted during metabolomics profiling which included the use of blanks, blind duplicates (5% of the BHS samples), and standard biochemical compounds which were integrated into every analyzed sample. Untargeted metabolomics profiling resulted in the detection and quantification of 1,466 metabolites. These included 956 known biochemical compounds in pathways related to amino acids (n=184), carbohydrates (n=25), cofactors and vitamins (n=34), energy (n=9), lipids (n=408), nucleotides (n=41), peptides (n=35), and xenobiotics (n=220). An additional 510 unnamed compounds currently lacking chemical standards were also quantified. These metabolites were labeled with an “X” followed by numbers (e.g., X-12345) and may be identified upon the eventual acquisition of a matching purified standard (or via classical structural analysis).

Prior to the statistical analysis, additional quality control and manipulation of the metabolite data was undertaken. Batch effects were assessed using principal components analysis, which revealed no evidence of clustering of metabolite data by run-days. Data filtering removed 213 metabolites that were missing or below the detection threshold in more than 80% of samples and 51 metabolites with a reliability coefficient <0.3 based on blind duplicate analysis. Among the 1,202 metabolites passing quality control, 167 were missing or below the detection threshold in 50% to 80% of the samples. Similar to previous analyses [45], these metabolites were analyzed as ordinal variables after categorization into one of three mutually exclusive groups: 1) missing or below-the-detection-limit; 2) below the median of detectable values; or 3) greater than or equal to the median of detectable values. The remaining 1,035 metabolites were analyzed as continuous variables, where the minimum observed value was imputed for metabolites with missing or below-the-detection-limit values.

### Measurement of study covariables

Covariable data were collected following stringent protocols that have been employed consistently at each clinical study visit [46]. Questionnaires were administered to obtain information on demographic characteristics (including age, gender, ethnicity, and education) and lifestyle risk factors (including cigarette smoking and alcohol consumption). Depression was assessed using the CES-D instrument [47], which has been validated previously and used extensively for research purposes [48,49]. Anthropometric measures were obtained by trained staff with participants in light clothing without shoes. During each visit, body weight and height were measured twice to the nearest 0.1 kg and 0.1 cm, respectively. The mean values of height and weight were used to estimate body mass index (BMI), which was calculated as weight in kilograms divided by height in square meters. Blood pressure (BP) was measured in the morning in triplicate by each of two trained observers using a mercury sphygmomanometer with the participant in a relaxed, sitting position. Systolic and diastolic BP levels were measured as the first and fifth Korotkoff sounds, respectively. The mean of the six BP values were used to estimate BP at each study visit.

Participants were instructed to fast for 12 hours prior to the blood sample collection. Serum total cholesterol (TC), high density lipoprotein cholesterol (HDL-C), and triglyceride (TG) levels were assayed using an enzymatic procedure as part of a lipid panel (Laboratory Corporation of America, Burlington, NC, USA) [50,51]. Low-density lipoprotein cholesterol (LDL-C) was calculated using the Friedewald equation (LDL-C = TC - HDL-C - TG/5) for those with TG less than 400 mg/dl [52]. Glucose was measured in adults using a multichemistry (SMA20) profile by enzymatic procedures using the multichannel Olympus Au-5000 Analyzer (Olympus, Lake Success, New York) [53].

### Measurement of cognition phenotypes

Global cognitive function and specific cognitive domains were assessed during the 2013-2016 visit using a battery of eight standard tests (Supplementary Figure 1). Tests were conducted by trained technicians and included the following: 1) Logical Memory I (WMS-IV), assessing narrative memory under a free recall condition; 2) Logical Memory II (WMS-IV), assessing long-term narrative memory with free recall; 3) Recognition (WMS-IV), assessing long-term narrative memory with recognition tasks; 4) Digit Span (WAIS-IV), assessing attention, working memory and executive function via two tasks (Digit Span Forward and Digit Span Backward); 5) Word and Letter Reading (WRAT-4), assessing decoding capability; 6) Vocabulary (WAIS-IV), assessing word knowledge and verbal concept formation; 7) Digit Symbol Coding (WAIS-IV), assessing processing speed and working memory; and 8) Trail Making Test (TMT), assessing visual search, scanning, speed of processing, mental flexibility, and executive function via two tasks (TMT forms A and B). For all tests except for the TMT, higher scores reflect better cognitive function. For the TMT, the opposite is true, with lower scores indicating better cognitive function.

To estimate global cognition, crude scores from the eight tests were Z-score transformed to a mean of 0 and standard deviation of 1. After flipping the sign of the TMT test, scores were summed for each study participant.

To normalize the distributions of the cognitive function phenotypes, crude scores were normalized using a rank-based inverse normal transformation. The transformed values were then rescaled to reflect the original trait distribution (multiplying by the original standard deviation), which should provide meaningful effect estimates in association analyses [54].

### Statistical analysis

Characteristics of study participants were presented as means and standard deviations (SDs) or median and interquartile range (IQRs) for continuous variables and as percentages for categorical variables.

### *Association of single metabolites with cognition phenotypes*

Multiple linear regression models were used to analyze the associations between each metabolite and cognition phenotype after adjustment for age, gender, ethnicity, cigarette smoking, drinking, education, depression, vocabulary, BMI, systolic BP, LDL-C, and glucose. Analyses were performed in the overall sample and according to both ethnicity and sex. A stringent Bonferroni correction for testing 1,202 metabolites was employed, corresponding to an α-threshold of 4.16×10^-5^ (0.05/1202). To minimize false positive findings, only metabolites achieving this p-value in any of the overall or ethnicity-sex specific analyses, and displaying consistent effect directions across all analyses, were considered statistically significant. Pairwise correlations of identified metabolites were assessed using Pearson correlation. All statistical analyses were performed in SAS (version 9.4; SAS Institute, Cary, NC) and in R (version 3.3.3).

### *Associations of metabolite modules with cognition phenotype*

To identify networks of highly correlated serum metabolites among BHS participants, weighted correlation network analysis (WGCNA) was utilized [55]. Unlike principal component analysis, this unsupervised data reduction technique allows for dependency between components, which may more accurately represent the related biological pathways of identified metabolites [55,56]. A description of WGCNA and its application to metabolomics studies has been reported previously [55,57]. Briefly, the metabolite network was constructed as an adjacency matrix based on the weighted pairwise-correlations of all metabolites [58]. Modules, defined as densely interconnected metabolites, were then identified from the network using an unsupervised hierarchical clustering approach [59]. For each module, an eigenmetabolite was generated. This measure represents the module’s first principal component and can be interpreted as its weighted average metabolite value. Because preliminary analyses revealed similar metabolite clustering across ethnic groups, metabolite modules were constructed using metabolite data for the 1,202 metabolites passing quality control among all study participants. To determine which biological pathways were best represented by each module, the metabolites most strongly correlated with each module’s eigenmetabolite (r>0.70) were identified, and the sub-pathways representing those metabolites were used to label each module.

Adjusted cognition phenotype measures were created using the residual values generated by regressing each raw cognition phenotype on age, gender, ethnicity, cigarette smoking, drinking, education, depression, vocabulary, BMI, SBP, LDL-C and glucose. The correlations between each module (eigenmetabolite) and the adjusted cognition phenotypes were then estimated in the overall sample and according to both ethnicity and sex. To correct for testing 9 serum metabolite modules (eigenmetabolites), a Bonferroni corrected α-threshold of 5.56×10^-3^ (0.05/9) was employed. Similar to the single metabolite study, modules achieving this p-value in any of the overall or ethnicity-sex specific analyses and demonstrating consistent effect directions across all analyses, were considered significant. These analyses were performed using the WGCNA package in R (version 3.3.3).

## Supplementary Material

Supplementary Figures

Supplementary Tables
