# Supplementary material for: Novel serum metabolites associate with cognition phenotypes among Bogalusa Heart Study participants
Source: Aging (Albany NY). 2019 Jul 21;11(14):5124–39. doi: 10.18632/aging.102107 (PMC6682535; doi:10.18632/aging.102107)
Supplement: Supplementary Figures [file aging-11-102107-s002.pdf]

SUPPLEMENTARY FIGURES

Supplementary Figure 1. Cognitive domains and the corresponding assessments

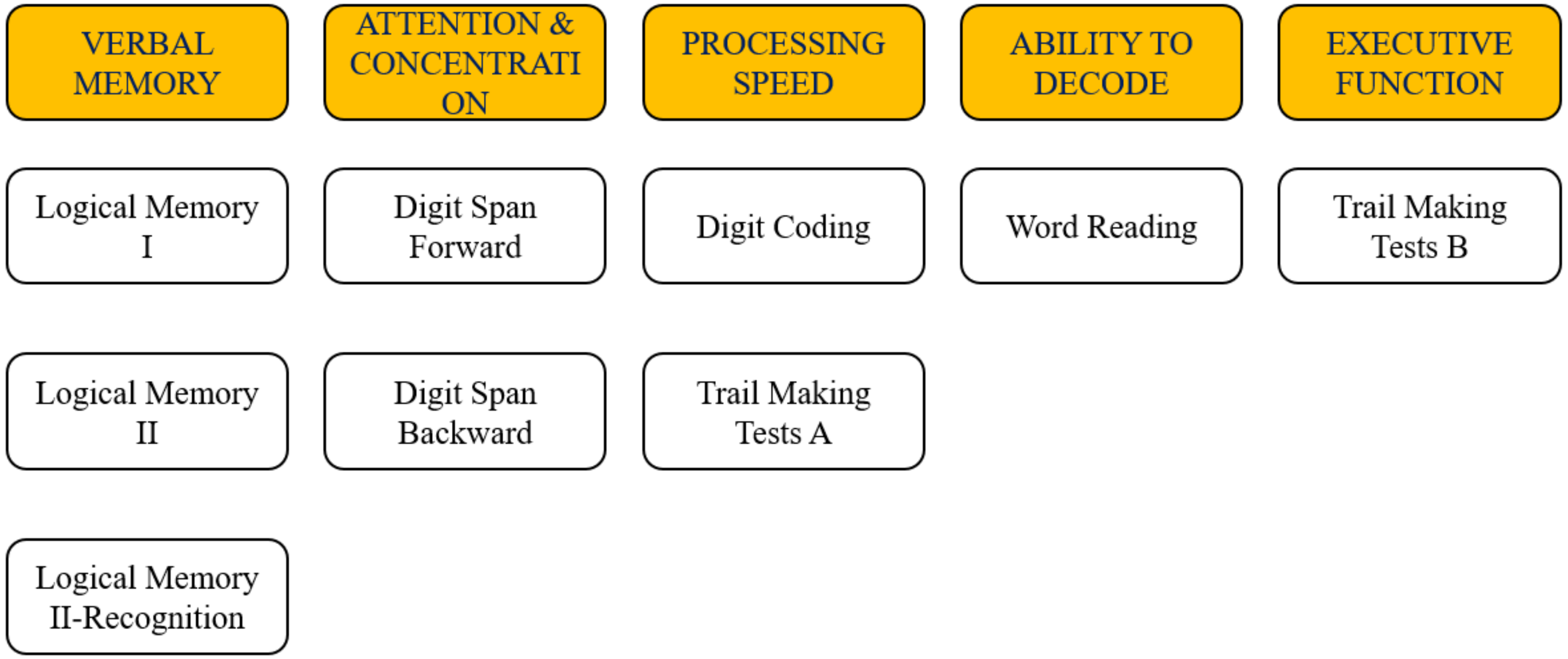

Supplementary Figure 2. Volcano plots of effect sizes versus –log10 P values for all 1202 metabolites among BHS participants, according to cognitive domain

2a) Verbal memory domain (logical memory I test)

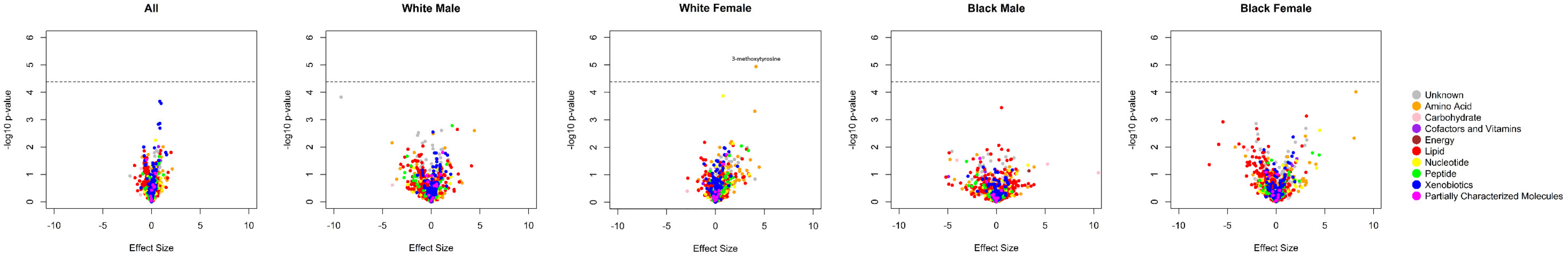

2b) Verbal memory domain (logical memory II test)

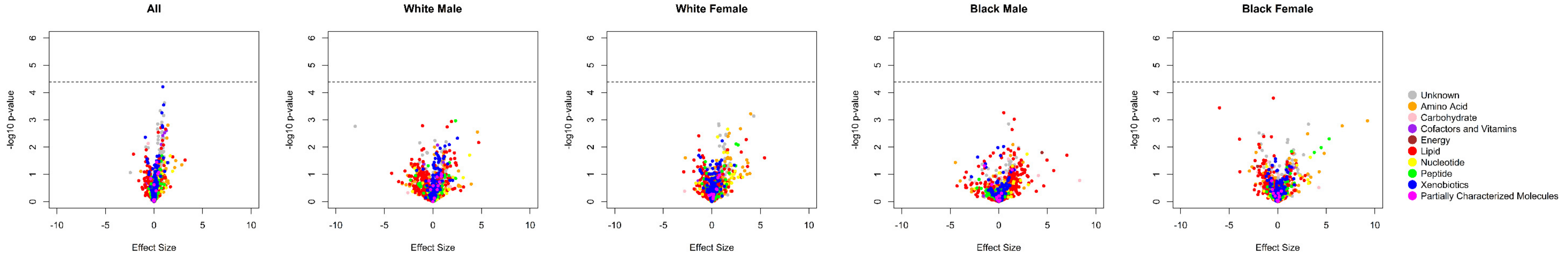

2c) Verbal memory domain (logical memory II-recognition)

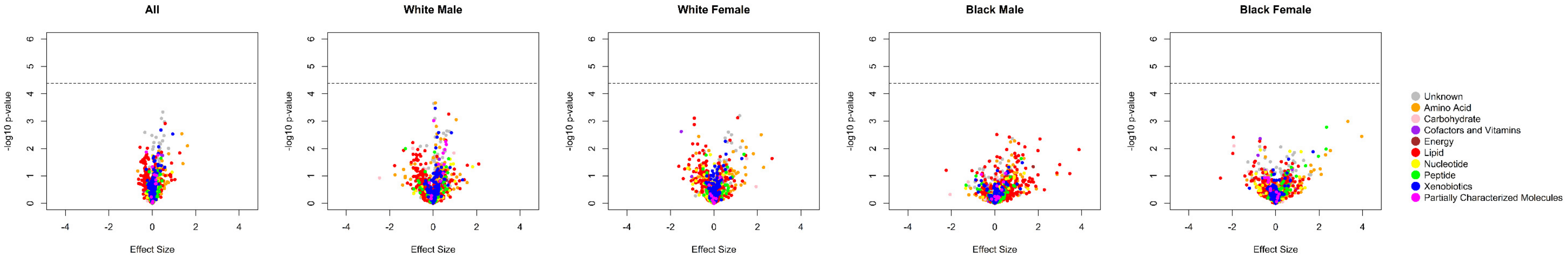

2d) Attention and concentration domain (digit span forward test)

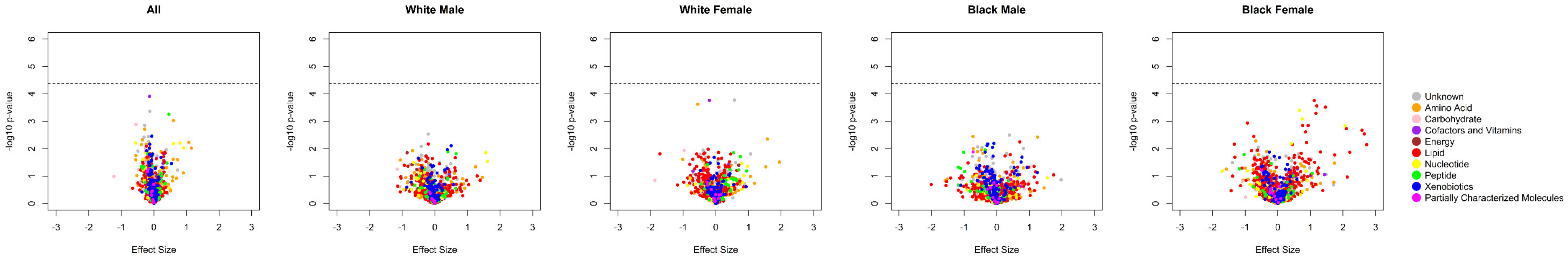

2e) Attention and concentration domain (digit span backward)

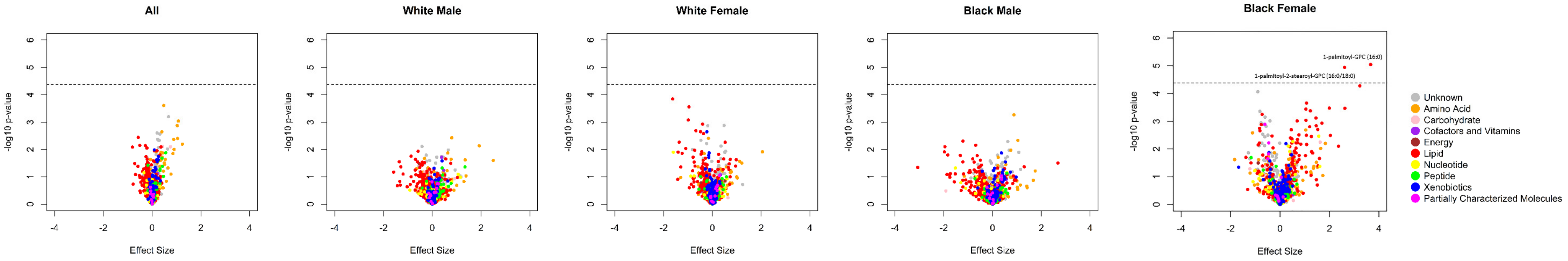

2f) Ability to decode domain (word reading test)

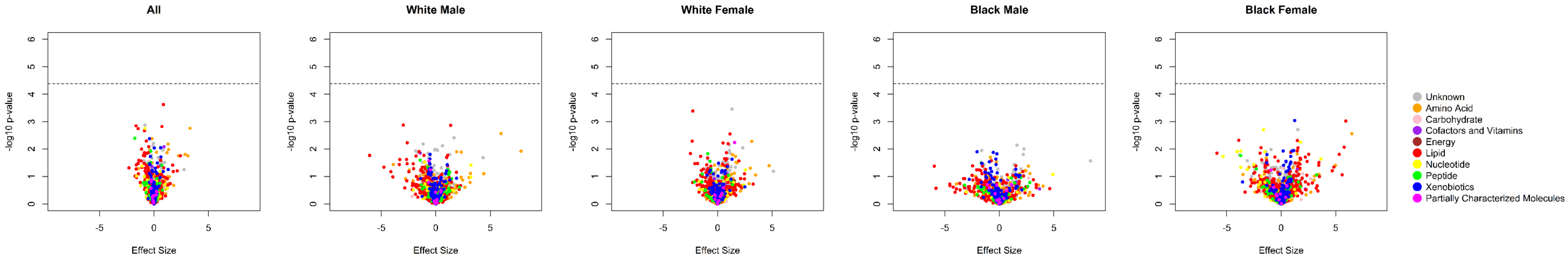

2g) Executive function domain (trail making test B)

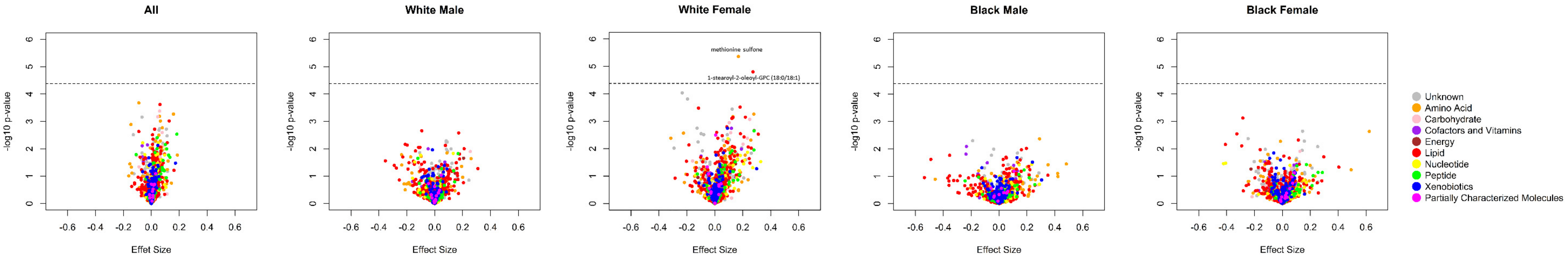

Supplementary Figure 3. Correlations of metabolite modules with cognition

3a) Verbal memory domain (logical memory I test)

Sub-pathways representing most highly correlated metabolites (r>0.7)	Overall r (P)	White Male r (P)	White Female r (P)	Black Male r (P)	Black Female r (P)
Lysophospholipid; Phosphatidylethanolamine (PE); Phosphatidylcholine (PC); Monoacylglycerol; Diacylglycerol	-0.029 (0.3)	-0.11 (0.06)	-0.006 (0.9)	-0.013 (0.9)	0.0034 (1)
Fatty acid metabolism (acyl choline); Lysophospholipid	-0.028 (0.3)	-0.073 (0.2)	0.015 (0.7)	-0.057 (0.5)	-0.039 (0.5)
Long chain fatty acid; Polyunsaturated fatty acid (n3 and n6); Fatty acid, monohydroxy; Fatty Acid, branched; Medium chain fatty acid; Endocannabinoid	-0.023 (0.4)	-0.069 (0.2)	-0.004 (0.9)	0.1 (0.2)	-0.11 (0.08)
Sphingolipid metabolism	-0.0035 (0.9)	-0.008 (0.9)	0.041 (0.4)	0.028 (0.7)	-0.1 (0.1)
Primary bile acid metabolism; Secondary bile acid metabolism	0.02 (0.5)	0.021 (0.7)	-0.028 (0.5)	0.11 (0.2)	-0.074 (0.2)
Methionine, cysteine, SAM and taurine, polyamine Metabolism; Tryptophan, tyrosine, lysine, histidine metabolism, and other amino acid; Aminosugar, pentose metabolism, and other carbohydrate; Ascorbate and aldarate, and tocopherol metabolism; Purine and pyrimidine Metabolism; Acetylated peptides; Mevalonate metabolism, fatty acid, dicarboxylate, and androgenic steroids; Food component, benzoate metabolism, and other xenobiotics	0.015 (0.6)	0.011 (0.9)	0.06 (0.2)	0.032 (0.7)	-0.074 (0.2)
Food component/Plant	0.0016 (1)	-0.059 (0.3)	-0.012 (0.8)	0.092 (0.3)	0.041 (0.5)
Androgenic steroids	0.031 (0.3)	-0.0069 (0.9)	0.086 (0.07)	-0.077 (0.4)	0.09 (0.2)
Androgenic steroids; Pregnenolone steroids; Progestin steroids	-0.012 (0.7)	-0.074 (0.2)	0.00057 (1)	0.031 (0.7)	0.016 (0.8)

Logical Memory I

3b) Verbal memory domain (logical memory II test)

Sub-pathways representing most highly correlated metabolites (r>0.7)	Overall r (P)	White Male r (P)	White Female r (P)	Black Male r (P)	Black Female r (P)
Lysophospholipid; Phosphatidylethanolamine (PE); Phosphatidylcholine (PC); Monoacylglycerol; Diacylglycerol	-0.016 (0.6)	-0.098 (0.08)	-0.013 (0.8)	0.12 (0.2)	-0.0082 (0.9)
Fatty acid metabolism (acyl choline); Lysophospholipid	-0.023 (0.4)	-0.061 (0.3)	-0.018 (0.7)	0.066 (0.4)	-0.051 (0.4)
Long chain fatty acid; Polyunsaturated fatty acid (n3 and n6); Fatty acid, monohydroxy; Fatty Acid, branched; Medium chain fatty acid; Endocannabinoid	-0.008 (0.8)	-0.059 (0.3)	-0.0053 (0.9)	0.17 (0.04)	-0.087 (0.2)
Sphingolipid metabolism	-0.01 (0.7)	0.00054 (1)	0.0097 (0.8)	-0.042 (0.2)	-0.045 (0.5)
Primary bile acid metabolism; Secondary bile acid metabolism	0.024 (0.4)	0.014 (0.8)	-0.0023 (1)	0.11 (0.2)	-0.092 (0.1)
Methionine, cysteine, SAM and taurine, polyamine Metabolism; Tryptophan, tyrosine, lysine, histidine metabolism, and other amino acid; Aminosugar, pentose metabolism, and other carbohydrate; Ascorbate and aldarate, and tocopherol metabolism; Purine and pyrimidine Metabolism; Acetylated peptides; Mevalonate metabolism, fatty acid, dicarboxylate, and androgenic steroids; Food component, benzoate metabolism, and other xenobiotics	0.044 (0.1)	0.0096 (0.9)	0.081 (0.09)	0.11 (0.2)	-0.017 (0.8)
Food component/Plant	0.0073 (0.8)	-0.025 (0.7)	-0.014 (0.8)	0.11 (0.2)	-0.00015 (1)
Androgenic steroids	0.049 (0.09)	0.055 (0.3)	0.062 (0.2)	-0.038 (0.6)	0.11 (0.08)
Androgenic steroids; Pregnenolone steroids; Progestin steroids	0.027 (0.4)	-0.0051 (0.9)	-0.0035 (0.9)	0.16 (0.05)	0.031 (0.6)

Logical Memory II

3c) Verbal memory domain (logical memory II-recognition test)

Sub-pathways representing most highly correlated metabolites (r>0.7)	Overall r (P)	White Male r (P)	White Female r (P)	Black Male r (P)	Black Female r (P)
Lysophospholipid; Phosphatidylethanolamine (PE); Phosphatidylcholine (PC); Monoacylglycerol; Diacylglycerol	-0.04 (0.2)	-0.091 (0.1)	-0.074 (0.1)	0.052 (0.5)	0.015 (0.8)
Fatty acid metabolism (acyl choline); Lysophospholipid	-0.016 (0.6)	-0.018 (0.7)	-0.079 (0.09)	0.042 (0.6)	0.057 (0.4)
Long chain fatty acid; Polyunsaturated fatty acid (n3 and n6); Fatty acid, monohydroxy; Fatty Acid, branched; Medium chain fatty acid; Endocannabinoid	-0.03 (0.3)	-0.066 (0.2)	-0.078 (0.1)	0.11 (0.2)	0.0014 (1)
Sphingolipid metabolism	0.013 (0.7)	0.041 (0.5)	-0.0024 (1)	0.16 (0.05)	-0.067 (0.3)
Primary bile acid metabolism; Secondary bile acid metabolism	0.0037 (0.9)	-0.054 (0.3)	-0.0042 (0.9)	0.093 (0.3)	-0.035 (0.6)
Methionine, cysteine, SAM and taurine, polyamine Metabolism; Tryptophan, tyrosine, lysine, histidine metabolism, and other amino acid; Aminosugar, pentose metabolism, and other carbohydrate; Ascorbate and aldarate, and tocopherol metabolism; Purine and pyrimidine Metabolism; Acetylated peptides; Mevalonate metabolism, fatty acid, dicarboxylate, and androgenic steroids; Food component, benzoate metabolism, and other xenobiotics	0.043 (0.1)	0.053 (0.3)	0.02 (0.7)	0.1 (0.2)	-0.062 (0.3)
Food component/Plant	-0.0059 (0.8)	-0.015 (0.8)	-0.043 (0.4)	0.083 (0.3)	-0.016 (0.8)
Androgenic steroids	0.038 (0.2)	0.06 (0.3)	0.041 (0.4)	-0.045 (0.6)	0.079 (0.2)
Androgenic steroids; Pregnenolone steroids; Progestin steroids	0.018 (0.5)	0.011 (0.8)	0.0076 (0.9)	0.046 (0.6)	0.011 (0.9)

Logical Memory II-Recognition

3d) Attention and concentration domain (digit span forward test)

Sub-pathways representing most highly correlated metabolites (r>0.7)	Overall r (P)	White Male r (P)	White Female r (P)	Black Male r (P)	Black Female r (P)
Lysophospholipid; Phosphatidylethanolamine (PE); Phosphatidylcholine (PC); Monoacylglycerol; Diacylglycerol	-0.042 (0.2)	-0.053 (0.3)	-0.086 (0.07)	-0.048 (0.6)	0.076 (0.2)
Fatty acid metabolism (acyl choline); Lysophospholipid	-0.014 (0.6)	-0.013 (0.8)	-0.11 (0.02)	-0.054 (0.5)	0.19 (0.002)
Long chain fatty acid; Polyunsaturated fatty acid (n3 and n6); Fatty acid, monohydroxy; Fatty Acid, branched; Medium chain fatty acid; Endocannabinoid	-0.00057 (1)	-0.0068 (0.9)	0.031 (0.5)	0.097 (0.2)	-0.1 (0.1)
Sphingolipid metabolism	-0.00097 (1)	0.014 (0.8)	-0.047 (0.3)	0.056 (0.5)	0.019 (0.8)
Primary bile acid metabolism; Secondary bile acid metabolism	-0.038 (0.2)	-0.056 (0.3)	0.01 (0.8)	-0.056 (0.5)	-0.099 (0.1)
Methionine, cysteine, SAM and taurine, polyamine Metabolism; Tryptophan, tyrosine, lysine, histidine metabolism, and other amino acid; Aminosugar, pentose metabolism, and other carbohydrate; Ascorbate and aldarate, and tocopherol metabolism; Purine and pyrimidine Metabolism; Acetylated peptides; Mevalonate metabolism, fatty acid, dicarboxylate, and androgenic steroids; Food component, benzoate metabolism, and other xenobiotics	-0.062 (0.03)	-0.09 (0.1)	0.0073 (0.9)	-0.098 (0.2)	-0.095 (0.1)
Food component/Plant	-0.011 (0.7)	-0.0083 (0.9)	0.026 (0.6)	-0.027 (0.7)	-0.062 (0.3)
Androgenic steroids	0.036 (0.2)	0.011 (0.8)	0.0029 (1)	0.14 (0.09)	0.092 (0.1)
Androgenic steroids; Pregnenolone steroids; Progestin steroids	-0.031 (0.3)	-0.078 (0.2)	0.067 (0.2)	-0.054 (0.5)	-0.11 (0.07)

Digit Span Forward

3e) Attention and concentration domain (digit span backward test)

Sub-pathways representing most highly correlated metabolites (r>0.7)	Overall r (P)	White Male r (P)	White Female r (P)	Black Male r (P)	Black Female r (P)
Lysophospholipid; Phosphatidylethanolamine (PE); Phosphatidylcholine (PC); Monoacylglycerol; Diacylglycerol	-0.025 (0.4)	-0.11 (0.06)	-0.06 (0.2)	-0.089 (0.3)	0.21 (0.001)
Fatty acid metabolism (acyl choline); Lysophospholipid	-0.042 (0.2)	-0.077 (0.2)	-0.15 (0.001)	-0.023 (0.8)	0.2 (0.001)
Long chain fatty acid; Polyunsaturated fatty acid (n3 and n6); Fatty acid, monohydroxy; Fatty Acid, branched; Medium chain fatty acid; Endocannabinoid	-0.042 (0.1)	-0.04 (0.5)	-0.015 (0.7)	-0.031 (0.7)	-0.1 (0.1)
Sphingolipid metabolism	1.7e-05 (1)	9e-04 (1)	0.019 (0.7)	-0.027 (0.7)	-0.028 (0.7)
Primary bile acid metabolism; Secondary bile acid metabolism	-0.037 (0.2)	-0.096 (0.08)	0.038 (0.4)	-0.061 (0.5)	-0.0064 (0.9)
Methionine, cysteine, SAM and taurine, polyamine Metabolism; Tryptophan, tyrosine, lysine, histidine metabolism, and other amino acid; Aminosugar, pentose metabolism, and other carbohydrate; Ascorbate and aldarate, and tocopherol metabolism; Purine and pyrimidine Metabolism; Acetylated peptides; Mevalonate metabolism, fatty acid, dicarboxylate, and androgenic steroids; Food component, benzoate metabolism, and other xenobiotics	-0.0064 (0.8)	-0.023 (0.7)	0.035 (0.5)	0.02 (0.8)	-0.088 (0.2)
Food component/Plant	-0.017 (0.6)	-0.045 (0.4)	0.024 (0.6)	-0.057 (0.5)	-0.0072 (0.9)
Androgenic steroids	0.044 (0.1)	0.068 (0.2)	-0.025 (0.6)	0.098 (0.2)	0.13 (0.04)
Androgenic steroids; Pregnenolone steroids; Progestin steroids	-0.01 (0.7)	-0.013 (0.8)	0.0039 (0.9)	-0.053 (0.5)	0.023 (0.7)

Digit Span Backward

3f) Ability to decode domain (word reading test)

Sub-pathways representing most highly correlated metabolites (r>0.7)	Overall r (P)	White Male r (P)	White Female r (P)	Black Male r (P)	Black Female r (P)
Lysophospholipid; Phosphatidylethanolamine (PE); Phosphatidylcholine (PC); Monoacylglycerol; Diacylglycerol	-0.048 (0.1)	-0.1 (0.07)	-0.079 (0.09)	-0.074 (0.4)	0.12 (0.05)
Fatty acid metabolism (acyl choline); Lysophospholipid	-0.041 (0.2)	-0.03 (0.6)	-0.072 (1)	-0.079 (0.3)	0.049 (0.4)
Long chain fatty acid; Polyunsaturated fatty acid (n3 and n6); Fatty acid, monohydroxy; Fatty Acid, branched; Medium chain fatty acid; Endocannabinoid	-0.026 (0.4)	-0.054 (0.3)	-0.0048 (0.9)	0.068 (0.4)	-0.089 (0.2)
Sphingolipid metabolism	0.0037 (0.9)	-0.069 (0.2)	0.044 (0.4)	0.047 (0.6)	-0.017 (0.8)
Primary bile acid metabolism; Secondary bile acid metabolism	-0.0093 (0.8)	0.00092 (1)	0.062 (0.2)	-0.099 (0.2)	0.033 (0.6)
Methionine, cysteine, SAM and taurine, polyamine Metabolism; Tryptophan, tyrosine, lysine, histidine metabolism, and other amino acid; Aminosugar, pentose metabolism, and other carbohydrate; Ascorbate and aldarate, and tocopherol metabolism; Purine and pyrimidine Metabolism; Acetylated peptides; Mevalonate metabolism, fatty acid, dicarboxylate, and androgenic steroids; Food component, benzoate metabolism, and other xenobiotics	-0.028 (0.3)	-0.035 (0.5)	0.031 (0.5)	-0.052 (0.5)	-0.048 (0.4)
Food component/Plant	-0.031 (0.3)	-0.11 (0.04)	-0.0033 (0.9)	-0.046 (0.6)	0.068 (0.3)
Androgenic steroids	0.044 (0.1)	0.073 (0.2)	0.014 (0.8)	0.1 (0.2)	0.022 (0.7)
Androgenic steroids; Pregnenolone steroids; Progestin steroids	-0.036 (0.2)	-0.051 (0.4)	-0.052 (0.3)	0.018 (0.8)	-0.0067 (0.9)

Word Reading

3g) Executive function domain (trail making test B)

Sub-pathways representing most highly correlated metabolites (r>0.7)	Overall r (P)	White Male r (P)	White Female r (P)	Black Male r (P)	Black Female r (P)
Lysophospholipid; Phosphatidylethanolamine (PE); Phosphatidylcholine (PC); Monoacylglycerol; Diacylglycerol	0.026 (0.4)	-0.021 (0.7)	0.1 (0.03)	-0.03 (0.7)	-0.03 (0.6)
Fatty acid metabolism (acyl choline); Lysophospholipid	-0.0095 (0.7)	0.042 (0.5)	0.03 (0.5)	-0.13 (0.1)	-0.05 (0.4)
Long chain fatty acid; Polyunsaturated fatty acid (n3 and n6); Fatty acid, monohydroxy; Fatty Acid, branched; Medium chain fatty acid; Endocannabinoid	0.026 (0.4)	0.076 (0.2)	0.031 (0.5)	0.03 (0.7)	-0.044 (0.5)
Sphingolipid metabolism	-0.0058 (0.8)	0.026 (0.6)	-0.021 (0.7)	-0.16 (0.05)	0.073 (0.2)
Primary bile acid metabolism; Secondary bile acid metabolism	0.074 (0.01)	0.022 (0.7)	0.1 (0.03)	0.12 (0.1)	0.087 (0.2)
Methionine, cysteine, SAM and taurine, polyamine Metabolism; Tryptophan, tyrosine, lysine, histidine metabolism, and other amino acid; Aminosugar, pentose metabolism, and other carbohydrate; Ascorbate and aldarate, and tocopherol metabolism; Purine and pyrimidine Metabolism; Acetylated peptides; Mevalonate metabolism, fatty acid, dicarboxylate, and androgenic steroids; Food component, benzoate metabolism, and other xenobiotics	0.064 (0.03)	-0.01 (0.9)	0.14 (0.004)	0.12 (0.2)	0.086 (0.2)
Food component/Plant	0.023 (0.4)	0.029 (0.6)	0.0099 (0.8)	0.043 (0.6)	0.021 (0.7)
Androgenic steroids	-0.013 (0.7)	-0.031 (0.6)	-0.0045 (0.9)	0.091 (0.3)	-0.093 (0.1)
Androgenic steroids; Pregnenolone steroids; Progestin steroids	0.044 (0.1)	0.027 (0.6)	0.083 (0.08)	0.13 (0.1)	-0.078 (0.2)

Trail Making Test B
