# Supplementary material for: Novel serum metabolites associate with cognition phenotypes among Bogalusa Heart Study participants
Source: Aging (Albany NY). 2019 Jul 21;11(14):5124–39. doi: 10.18632/aging.102107 (PMC6682535; doi:10.18632/aging.102107)
Supplement: Supplementary Tables [file aging-11-102107-s001.pdf]

SUPPLEMENTARY TABLES

Supplementary Table 1. Unknown metabolites achieving significant in BHS participants.

Super Pathway	Sub Pathway	Metabolite	Overall			White Male			White Female			Black Male			Black Female		
			ES	SE	P	ES	SE	P	ES	SE	P	ES	SE	P	ES	SE	P
Processing Speed																	
Unknown	Unknown	X_21840[1]	2.37	0.51	4.32E-06	1.50	0.92	1.03E-01	3.33	0.90	2.55E-04	3.06	1.30	1.99E-02	2.60	1.12	2.05E-02

ES=Effect size; SE=Standard error. Adjusted by age, gender, ethnicity, cigarette smoking, drinking, education, depression, vocabulary, BMI, SBP, LDL-C and glucose.

1. Associated with digit coding test.

Supplementary Table 2. Metabolites with inconsistent effect directions in BHS participants.

Super Pathway	Sub Pathway	Metabolite	Overall			White Male			White Female			Black Male			Black Female		
			ES	SE	P	ES	SE	P	ES	SE	P	ES	SE	P	ES	SE	P
Global Cognition																	
Lipid	Fatty Acid, Monohydroxy	3-hydroxyoctanoate[1]	-0.05	0.13	7.08E-01	0.19	0.22	4.01E-01	0.01	0.20	9.49E-01	0.12	0.41	7.78E-01	-1.90	0.44	2.21E-05
Lipid	Phospholipid Metabolism	phosphoethanolamine[1]	0.65	0.31	3.57E-02	0.22	0.59	7.09E-01	0.15	0.56	7.88E-01	-0.19	0.73	7.91E-01	2.77	0.62	1.21E-05
Verbal Memory																	
Amino Acid	Tyrosine Metabolism	3-methoxytyrosine[2]	1.19	0.46	9.76E-03	0.68	1.20	5.72E-01	4.14	0.93	1.16E-05	-0.26	1.50	8.61E-01	0.40	0.63	5.26E-01
Attention &Concentration																	
Lipid	Lysophospholipid	1-palmitoyl-GPC (16:0)[3]	-0.06	0.40	8.75E-01	0.08	0.86	9.25E-01	-1.30	0.64	4.41E-02	-1.87	1.07	8.29E-02	3.66	0.81	9.01E-06
Lipid	Phosphatidylcholine (PC)	1-palmitoyl-2-stearoyl-GPC (16:0/18:0)[3]	-0.32	0.27	2.28E-01	-0.87	0.53	1.00E-01	-1.09	0.42	9.34E-03	-1.10	0.72	1.32E-01	2.61	0.58	1.14E-05
Processing Speed																	
Lipid	Plasmalogen	1-(1-enyl-palmitoyl)-2-palmitoleoyl-GPC (P-16:0/16:1)*[4]	0.07	0.02	1.39E-05	0.08	0.03	1.46E-02	0.09	0.03	8.29E-04	-0.02	0.06	7.11E-01	0.08	0.04	4.36E-02
Executive Function																	
Amino Acid	Methionine, Cysteine, SAM and Taurine Metabolism	methionine sulfone[5]	0.08	0.03	1.71E-03	-0.01	0.05	8.24E-01	0.17	0.04	4.38E-06	-0.01	0.07	8.67E-01	0.01	0.08	8.42E-01
Lipid	Phosphatidylcholine (PC)	1-stearoyl-2-oleoyl-GPC (18:0/18:1)[5]	0.08	0.04	5.94E-02	-0.04	0.07	5.61E-01	0.27	0.06	1.59E-05	-0.13	0.13	3.22E-01	-0.06	0.12	6.33E-01

ES=Effect size; SE=Standard error. Adjusted for age, gender, ethnicity, cigarette smoking, drinking, education, depression, vocabulary, BMI, SBP, LDL-C and glucose.

* Indicates compounds that have not been officially confirmed based on a standard, but we are confident in its identity.

1. Associated with global cognition score.
2. Associated with logical memory I test.
3. Associated with digit span backward test.
4. Associated with trail making test A test.
5. Associated with trail making test B test.
